# Shining a Light on Race: Contrast and Assimilation Effects in the Perception of Skin Tone and Racial Typicality

**DOI:** 10.3389/fpsyg.2020.604617

**Published:** 2020-11-27

**Authors:** Kevin R. Brooks, Daniel Sturman, O. Scott Gwinn

**Affiliations:** ^1^Department of Psychology, Faculty of Medicine, Health and Human Sciences, Macquarie University, Sydney, NSW, Australia; ^2^Perception in Action Research Centre (PARC), Faculty of Medicine, Health and Human Sciences, Macquarie University, Sydney, NSW, Australia; ^3^School of Psychology, Faculty of Health and Medical Sciences, The University of Adelaide, Adelaide, SA, Australia; ^4^School of Psychology, Flinders University, Adelaide, SA, Australia

**Keywords:** skin tone, facial morphology, race, assimilation, contrast, face perception, skin tone bias, lightness and brightness illusions

## Abstract

Researchers have long debated the extent to which an individual’s skin tone influences their perceived race. Brooks and Gwinn (2010) demonstrated that the race of surrounding faces can affect the perceived skin tone of a central target face without changing perceived racial typicality, suggesting that skin lightness makes a small contribution to judgments of race compared to morphological cues (the configuration and shape of the facial features). However, the lack of a consistent light source may have undermined the reliability of skin tone cues, encouraging observers to rely disproportionately on morphological cues instead. The current study addresses this concern by using 3D models of male faces with typically Black African or White European appearances that are illuminated by the same light source. Observers perceived target faces surrounded by White faces to have darker skin than those surrounded by Black faces, particularly for faces of intermediate lightness. However, when asked to judge racial typicality, a small assimilation effect was evident, with target faces perceived as more stereotypically White when surrounded by White than when surrounded by Black faces at intermediate levels of typicality. This evidence of assimilation effects for perceived racial typicality despite concurrent contrast effects on perceived skin lightness supports the previous conclusion that perceived skin lightness has little influence on judgments of racial typicality for racially ambiguous faces, even when lighting is consistent.

## Introduction

Considerations of perceived race can have critical consequences in daily life (Pavkov et al., 1989; Blair et al., 2002; Maddox and Gray, 2002; Eberhardt et al., 2006). Although academics from a variety of sub-disciplines have studied these consequences, relatively few studies have attempted to examine the visual cues to race and the way our brains use this information to determine perceived race and racial typicality.

**FIGURE 1:**
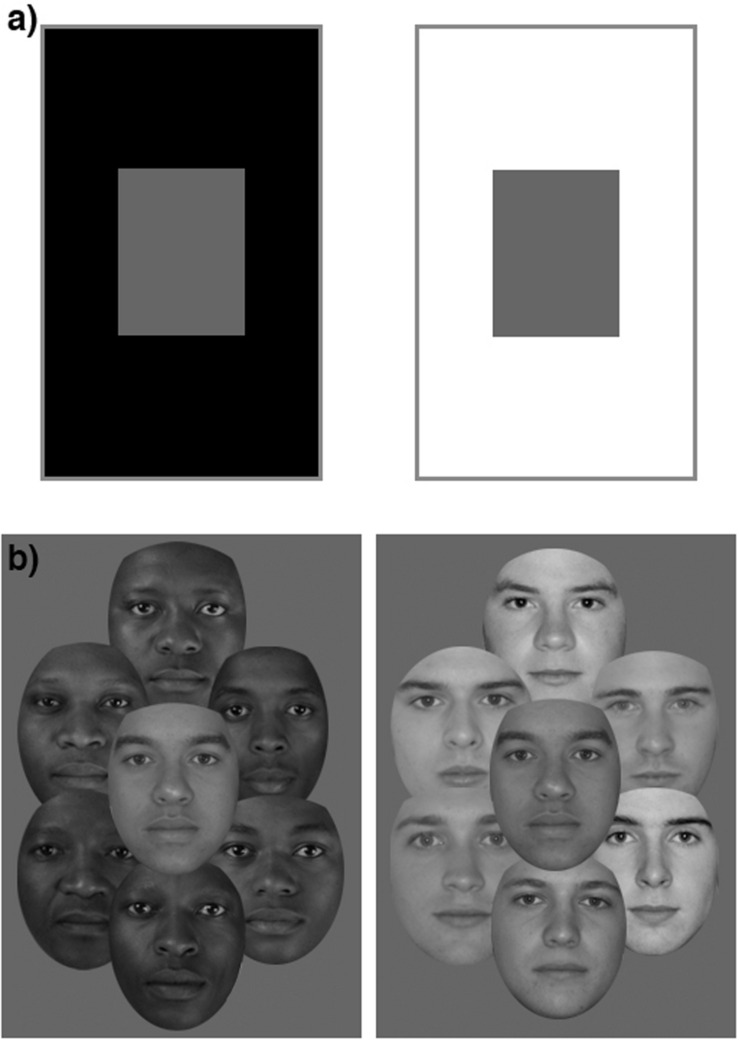
**(a)** The traditional lightness contrast illusion. **(b)** The face version of the lightness contrast illusion, as presented by Brooks and Gwinn (2010).

Faces of individuals belonging to some different racial groups, for example, those originating from sub-Saharan Africa compared to those from north western Europe, can show consistent differences in both their morphological shape (Hajnis et al., 1994) and skin reflectance, or “lightness” information (Jablonski and Chaplin, 2000). When asked to judge the relative importance of shape and skin cues in defining someone’s race, people tend to emphasize the contributions made by skin cues over shape cues (Brown et al., 1998). Indeed, the racial labels “Black” and “White” that are often used to describe people with African and European heritage would seem to reflect this belief. However, more explicit tests of the perceived race of facial images have indicated that skin cues may not be as important as is commonly believed (Hill et al., 1995; Stepanova and Strube, 2009; Brooks and Gwinn, 2010; Willenbockel et al., 2011; Gwinn and Brooks, 2015). In one of the earliest studies to reach this conclusion, Brooks and Gwinn (2010) attempted to influence the perceived race of faces using the well-known lightness contrast illusion (Agostini and Galmonte, 2002; Economou et al., 2007; Zeman et al., 2015). The traditional lightness contrast illusion figure involves a mid-gray rectangle, surrounded either by a black or by a white region (Figure 1a). Despite their identical photometric properties, the rectangle surrounded by the black region usually appears lighter than when viewed in isolation, while the rectangle with the white surround appears darker. For Brooks and Gwinn’s face version (Figure 1b), an equivalent result emerged. Surrounding a target face with Black faces or White faces resulted in the skin tone of the target face appearing lighter or darker, respectively. However, the perceived racial typicality of the target face was not affected, leading the authors to conclude that perceived skin tone has little or no influence on race judgments (Brooks and Gwinn, 2010). The same conclusion has been reached in other studies using various paradigms (Hill et al., 1995; Stepanova and Strube, 2009; Willenbockel et al., 2011; Gwinn and Brooks, 2015). Stepanova and Strube (2009) showed that although the contribution made by skin tone can increase when color images are used, morphology still accounts for a larger proportion of the variance in perceptions of racial typicality (although see Stepanova and Strube, 2012 for contradictory findings, especially for participants with more negative implicit racial attitudes). The contribution of skin tone can also increase when normal face processing is disrupted through inversion or short presentation durations (Willenbockel et al., 2011; Sun and Balas, 2012), or when children are used as participants (Balas et al., 2015; Dunham et al., 2015).

Although it may at first appear trivial, the process of judging of the lightness (i.e., reflectance, or “albedo”) of any surface, including skin, is complex. Rather than simply involving an assessment of the intensity of light rebounding from the surface and entering the eye (the luminance), accurate lightness perception must also adjust for the properties of the original light source (the illuminant) or sources (Alhazen, 1083/1989; von Helmholtz, 1866/1925; Gilchrist et al., 1999). Certainly, under the same illuminant, a white surface reflects a higher proportion of the incident light than a black surface, but a change in illuminant intensity can drastically affect the luminance of either, such that a brightly lit black surface can have a higher luminance than a dimly lit white surface. When the intensity of the illuminant can be accurately judged, its effects can be discounted and lightness can be veridically perceived, but this may not be possible when the properties of the light source are hard to discern. While the exact method of judging illuminant properties remains contentious, the mechanism must involve consideration of the luminance of other visible objects in the same scene (Gilchrist et al., 1999; Logvinenko and Tokunaga, 2011). It is also important to know which of the visible surfaces share the same illumination (Gilchrist, 2015). As an extreme example, when it is not clear which surfaces are lit by the same source, and the illuminant’s properties are incorrectly judged, a black surface can actually appear white (Gelb, 1929; cited in Gilchrist, 2015).

Many studies of face perception, including some of those cited above (e.g., Brooks and Gwinn, 2010; Willenbockel et al., 2011; Gwinn and Brooks, 2015), use 2D images of faces that have been tightly cropped, often excluding external facial features. This is likely to compromise the observer’s ability to judge the properties of the illuminant. In addition, Brooks and Gwinn (2010) presented several such stimuli overlapping each other, making it implausible that the cut-out faces are physically present in the same environment. Hence, the lighting conditions under which each face is being viewed are unclear, causing uncertainty as to whether differences in the brightness of the faces are the result of different illuminants or differences between the reflectance properties of the skin. The confusion may result in skin tone being deemed unreliable and therefore given little weight when making racial typicality judgments. If this were the case, the result would be an underestimation of the real-world contribution of skin tone in judgments of race. In the current study, we sought to address this shortcoming of the Brooks and Gwinn (2010) study by using 3D head models that more clearly occupy the same physical space and are illuminated under the same lighting conditions. Using the same paradigm as Brooks and Gwinn (2010), we now re-examine whether changes in perceived skin tone have a greater influence on perceptions of racial typicality under conditions of consistent lighting.

## Materials and Methods

### Participants

Thirty-two (nine male, 23 female) predominantly White^1^ undergraduate students enrolled in Psychology at Macquarie University, with a mean age of *M* = 20.1 (*SD* = 1.9) took part in the experiment. The number of participants was predetermined, based on Brooks and Gwinn (2010), which also had 32 participants. Participants gave informed consent and received course credit for enrolling in the study. The study was approved by the Macquarie University Faculty of Human Sciences Human Research Ethics Committee (Code: 5201400561), and conducted in accordance with the Declaration of Helsinki. Optical corrections were worn if necessary.

### Stimuli

**FIGURE 2:**
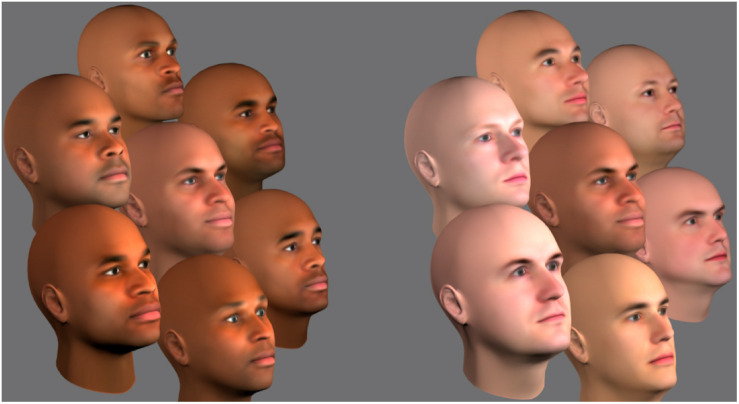
A central face at the 50% morph level embedded in a surround of Black faces **(left)** and White faces **(right)**.

The face images used in the study were originally produced and validated by Matheson and McMullen (2011) using FaceGen Modeller. For our purposes, 22 male faces were selected, 11 with appearances previously validated as being perceived as typically Black and 11 with appearances perceived as typically White (Matheson and McMullen, 2011). Five faces from each group (10 total) were selected to serve as “target” faces. Each White target face was paired with a Black target face and combined in a linear fashion using FaceGen to create five morphed images for each pair (25 total) that varied in the contribution made by the morphology and skin reflectance properties of the White (relative to the Black) face from 0 to 100% in steps of 25%. Using Blender 3D, the remaining six faces from each racial group (12 total) were used to create a White surround and a Black surround. Each target face was then separately embedded in a White, and in a Black surround (see Figure 2). Five different surrounds were created for each racial group by rearranging the position of the faces, resulting in the five target faces at each morph level appearing in unique surrounds. In Blender 3D, three diffuse spot lights of equal intensity illuminated the array of faces, one directly in front, one 30° to the left, and one 30° to the right. The camera was placed at a 45° angle, to create a three-quarter view of the faces. The final 50 images (5 morph levels × 5 target faces × 2 surrounds) measured 540 × 540 pixels and were presented in color on a Sony Trinitron G520/Dell P1130 CRT monitor.

### Procedure

The procedure used was the same as described in Brooks and Gwinn (2010). Images were presented using custom software created in Matlab 2013b (Mathworks, United States). At the beginning of each trial, an image was displayed on the screen for 3 s, following which participants were required to make a rating of the central face. In separate blocks, participants were required to rate the central face either in terms of the lightness of its skin or its racial typicality. The order of blocks was counterbalanced across participants. Ratings were provided using the mouse to move a slider further left or right on the screen. For typicality blocks, in which participants were explicitly instructed to make ratings of racial typicality, the left end of the scale was labeled “Stereotypically White” and the right labeled “Stereotypically Black.” For lightness blocks, in which participants were explicitly instructed to make ratings of skin lightness, the left end of the scale was simply labeled “White” and the right labeled “Black.” There was no time limit on responding or on the number of times participants could adjust their response before clicking the “Confirm” button at the bottom of the display. Once a response was confirmed, it was converted into a value between 0 and 100 (inclusive) for analysis. In separate trials, participants rated each target face when surrounded by White faces and when surrounded by Black faces. The order of image presentation was pseudo-randomized within each block, ensuring that a minimum of 10 trials were included in between the same target face being seen in each surround.

## Results

**FIGURE 3:**
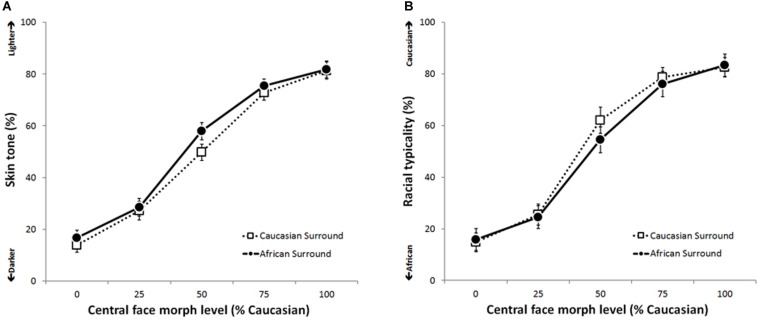
Mean skin lightness ratings **(A)** and racial typicality ratings **(B)** for the five morph levels when surrounded by either White or Black faces. Error bars show 95% CIs.

For each participant, an average score was calculated for each morph level when the target face was surrounded by White faces, and when surrounded by Black faces. Figure 3 shows mean skin lightness and typicality ratings for the five morph levels, plotted separately for the two surrounds. For either ratings of skin lightness (Figure 3A) or of racial typicality (Figure 3B), it is clear that increasing the morph level increases the ratings. These effects are entirely as expected, and will not be discussed in detail. More importantly, the surrounding faces appear to cause differing patterns of results for the two tasks. For skin lightness ratings, within each morph level, central faces appear to be rated as having darker skin when surrounded by White faces and lighter skin when they are surrounded by Black faces. However, for racial typicality, central faces appear to be rated as more typically White when surrounded by White faces versus Black faces. Although differences are small at other morph levels, these observations seem particularly clear at 50%. Statistical significance was formally assessed using two 2 × 5 repeated measures ANOVAs, with the factors Surround (White vs. Black) and Morph Level (0, 25, 50, 75, and 100%). Mauchly’s test indicated that the assumption of sphericity had been violated for the factor of Morph Level in both the ANOVAs analyzing ratings of skin lightness (χ^2^_9_ = 77.72, *p* < 0.0005) and racial typicality (χ^2^_9_ = 86.71, *p* < 0.0005), and so output from multivariate tests are reported.

For ratings of skin lightness, ANOVA revealed a significant main effect of Surround (*F*_1,31_ = 15.61, *p* < 0.0005, $\eta_{\text{p}}^{2}$ = 0.36) and of Morph Level (*F*_4,28_ = 247.33, *p* < 0.0005, $\eta_{\text{p}}^{2}$ = 0.97). Mean ratings showed that central faces were perceived as having darker skin when surrounded by White faces (*M* = 49, *SD* = 5.31) compared to when surrounded by Black faces (*M* = 52.1, *SD* = 6.12). A significant Surround × Morph Level interaction was also observed (*F*_4,28_ = 7.43, *p* < 0.0005, $\eta_{\text{p}}^{2}$ = 0.52), perhaps reflecting the earlier observation that the effect of Surround appears more pronounced at the 50% morph level compared to the other levels. To further examine this observation, follow-up comparisons were performed using five paired-sample *t*-tests (two-tailed). Using a Bonferroni correction, the critical alpha was adjusted to α = 0.01. These comparisons confirmed that at the 50% morph level, central faces were perceived as having significantly darker skin when surrounded by White faces (*M* = 49.78, *SD* = 9.16) compared to when surrounded by Black faces (*M* = 57.98, *SD* = 8.81) (*t*_31_ = 5.11, *p* < 0.0005, *d* = 0.91). At the 0% morph level, the central faces were also perceived as having significantly darker skin when surrounded by White faces (*M* = 13.84, *SD* = 7.4) compared to Black faces (*M* = 16.76, *SD* = 7.92) (*t*_31_ = 3.22, *p* = 0.003, *d* = 0.38). This effect was not significant at any other morph levels: 25% (*t*_31_ = 1, *p* = 0.324, *d* = 0.15), 75% (*t*_31_ = 2.67, *p* = 0.012, *d* = 0.35), 100% (*t*_31_ = 0.61, *p* = 0.55, *d* = 0.05).

For ratings of typicality, ANOVA revealed a significant main effect of Surround (*F*_1,31_ = 4.2, *p* = 0.049, $\eta_{\text{p}}^{2}$ = 0.12) and of Morph Level (*F*_4,28_ = 229.65, *p* < 0.0005, $\eta_{\text{p}}^{2}$ = 0.97). Mean ratings showed that central faces appeared more stereotypically White when surrounded by White faces (*M* = 52.74, *SD* = 6.93) compared to when surrounded by Black faces (*M* = 50.87, *SD* = 7.67). A significant Surround × Morph level interaction was also observed (*F*_4,28_ = 5.66, *p* = 0.002, $\eta_{\text{p}}^{2}$ = 0.48), presumably reflecting a more pronounced effect of Surround at the mid 50% morph level. Again, follow-up comparisons were performed using five paired-sample *t*-tests (two-tailed), and the critical alpha adjusted to a Bonferroni-corrected value of α = 0.01. These comparisons confirmed that at the 50% morph level, central faces were perceived as more stereotypically White when surrounded by White faces (*M* = 62.04, *SD* = 11.88) compared to when surrounded by Black faces (*M* = 54.51, *SD* = 12.15) (*t* = 3.98, *p* < 0.0005, *d* = 0.63). However, this effect was not significant at any other morph levels: 0% (*t*_31_ = 0.99, *p* = 0.328, *d* = 0.12), 25% (*t*_31_ = 0.58, *p* = 0.568, *d* = 0.12), 75% (*t*_31_ = 2.32, *p* = 0.027, *d* = 0.27), 100% (*t*_31_ = 0.82, *p* = 0.417, *d* = 0.09).

## Discussion

We examined whether the perception of skin lightness and racial typicality for face stimuli is affected by the properties of the faces surrounding them. In particular, we sought to establish whether the previous results of Brooks and Gwinn (2010) were artifacts of a highly artificial stimulus display, which may have degraded skin lightness information. For judgments of skin lightness, a contrast effect was found, with central faces appearing darker when surrounded by White faces compared to when surrounded by Black faces. However, for judgments of racial typicality, a small assimilation effect was found, with a racially ambiguous central target face being perceived as more stereotypically White when surrounded by White faces than when surrounded by Black faces. For both judgments, these effects were most pronounced for central faces that were racially ambiguous morphs comprising equal input from the White and Black component faces. Although we have no *a priori* reason to hypothesize that different results would be demonstrated for female stimuli, as we used only male faces this has yet to be confirmed.

Results from the skin tone judgments in the current study compliment those previously reported by Brooks and Gwinn (2010), and in experiment 3 of Sun and Balas (2012). That a face’s skin can be made to appear lighter or darker without also making that face appear more typically White or Black, respectively, accords with other evidence indicating that perceptions of race are largely driven by morphological shape cues and not skin reflectance cues (Hill et al., 1995; Brooks and Gwinn, 2010; Willenbockel et al., 2011; Gwinn and Brooks, 2015). However, an important difference between previous studies and the current study is the stimuli used. In previous studies, the facial images were tightly cropped following the jaw and hairlines, making it unclear whether all images were occupying the same environment and were illuminated by the same light source. This ambiguity of the illuminant may have resulted in observers deeming the reflectance information to be unreliable, as it is not clear whether the differences between the luminance of the skin in these photographs were the result of different reflectance properties of the surfaces, or differences in the illuminants. In the current study, we used 3D head models that more clearly occupy the same space and that appear under a uniform light source, making the skin surface lightness cues more reliable. Given the same light source, luminance differences must have resulted from differences in reflectance. Yet even under these conditions we found that skin cues still contribute relatively little to perceptions of race.

While the principal improvement of the current study over Brooks and Gwinn (2010) involved the consistent lighting environment, it should be acknowledged that this was not the only difference. In addition, the use of color full head models, as opposed to grayscale cropped photographs, may potentially have affected the ratings. While photographs often have the advantage of increased realism, cropping and reduction to grayscale significantly reduces any such benefit. Further, the head model stimuli used in this study have previously been validated in terms of realism and racial typicality by a previous study (Matheson and McMullen, 2011). Another difference is the viewpoint of the faces, presented in three-quarter view in the current study, compared to frontal images used in Brooks and Gwinn (2010). Interestingly, Hill et al. (1995) presented evidence that shape information is more likely to be prioritized over “texture” information in three-quarter compared to frontal view. However, Hill et al.’s task involved binary race categorization of Asian vs. Caucasian faces when shape and texture cues had been deliberately mismatched – a very different situation to ours. Regardless of these differences, the pattern of results for the two studies remains similar. Although it is not impossible that one of these differences influenced the results in one direction while another difference canceled this influence, a more parsimonious explanation is that none of them had any great influence in terms of judgments of lightness or racial typicality.

A minor difference between the current results and those previously reported by Brooks and Gwinn (2010) is the effect of the surrounding faces on the perceived racial typicality of the central faces. Whereas Brooks and Gwinn (2010) reported that the racial typicality of the central faces was not affected by the race of the surrounding faces, in the current study we found evidence of a small assimilation effect. That is, racially ambiguous (i.e., 50% morph level) central faces were perceived as being more stereotypically White when surrounded by White faces than when surrounded by Black faces. It is worth noting that this assimilation effect occurred despite the central faces being subject to skin lightness contrast effects, further supporting the conclusion that skin cues make a relatively small contribution to perceptions of race when racial typicality is ambiguous. Assimilation effects for race have previously been reported by Sun and Balas (2012), who suggested that they may be occurring as a result of ensemble coding (Ariely, 2001; Haberman and Whitney, 2009). Ensemble coding refers to instances in which observers rapidly extract the mean properties from a group of similar objects. For example, when shown images of faces expressing varying degrees of happiness or sadness, observers can later identify a face displaying the mean expression of the previously viewed set of faces (Haberman and Whitney, 2009). In cases of racial assimilation, observers may be extracting the mean race of each group of faces and applying that mean to the target image (Sun and Balas, 2012). The extraction of “average race” from a collection of face stimuli through ensemble coding has recently been demonstrated by Thornton et al. (2019).

Although assimilation effects for judgments of race are reported here and by Sun and Balas (2012), the nature of these effects is quite different between the two studies. In the latter study, assimilation effects in experiment 1 occurred only when the surround faces were White, and had been inverted – a manipulation known to disrupt normal face processing (Farah et al., 1995; Kanwisher et al., 1998; Freire et al., 2000). In experiment 2, when the properties of surround faces were manipulated independently, moprhology elicited a contrast effect, while skin tone appeared to be assimilative. Again, larger effects were shown for inverted surround faces. This pattern of results is clearly at odds with the assimilation effects on perceived race reported here, which occurred with upright surround faces even when morphology and skin tone cues were consistent. Obvious differences between the two studies lie in the geometric layout of the stimuli, the task used, and the details of the timing. Although the current study and the original Brooks and Gwinn (2010) study used overlapping face stimuli, Sun and Balas (2012) used stimuli that were physically separated. While this matter has yet to be formally investigated, smaller separations may allow us to predict greater assimilation effects for higher-level properties such as perceived race, given that the receptive fields of face-sensitive neurons, although large, are not limitless (Zhao and Chubb, 2001). The specific details of the experimental task may also influence whether contrast or assimilation effects occur. In the current study, judgments were made using a continuous sliding scale, whereas in Sun and Balas (2012), responses were binary. A further distinction involves stimulus presentation times: 3 s in the current study compared to 500 ms for Sun and Balas (2012). It may be that these presentation durations encourage different forms or levels of encoding, or exert other influences on racial categorization. In support of this suggestion, Sun and Balas (2012) also used a delayed matching task in a later experiment, finding a contrast effect for skin cues.

It is interesting to note that in studies of lightness perception using abstract stimuli, contrast and assimilation effects can be shown in the same kinds of displays with subtle changes of stimulus details and experimental procedure (Helson and Rholes, 1959; Beck, 1966; Steger, 1969). Conditions that tend to support contrast effects involve focused attention toward the stimulus, e.g., judgments concerning “figure” rather than “ground”; stimuli with longer durations; and more demanding tasks (Festinger et al., 1970). While pairwise comparisons favor assimilation, matching tasks are more likely to produce contrast effects. It may be noteworthy that in Sun and Balas (2012), a change of task from binary race categorization (experiments 1 and 2) to delayed matching (experiment 3) caused a change from assimilation to contrast. It is also worth considering whether differences between the two tasks’ results in the present study could be accounted for in this way. While lightness perception and racial typicality judgments involve identical stimulus durations and response interfaces/protocols, it could be argued that typicality judgments may require less explicit attention to the lightness of the skin, and hence be more conducive to lightness assimilation effects. This assimilative bias could then implicitly affect racial categorization, that is, if lightness did have an influence over perceived race. However, this suggestion appears speculative at best. Festinger et al.’s (1970) proposal that attention underlies the difference between lightness contrast and assimilation effects relies on object attention, which in this case would still need to be explicitly directed at the central face even in the typicality task. In this instance, only featural attention (to the lightness aspects of the face) would have been weakened. To our knowledge, manipulations of featural attention have never been shown to reverse lightness contrast effects.

## Conclusion

Using 3D head models with a uniform light source, we have addressed concerns regarding the perceived reliability of skin reflectance cues to race that were present in previous studies. The data demonstrate that surround faces can cause a contrast effect on apparent skin tone that does not emerge in judgments of racial typicality – the same general pattern of results shown by Brooks and Gwinn (2010). Furthermore, we observed a small assimilation effect for racial typicality that may represent a form of ensemble coding. Overall, this suggests that greater weight is given to shape cues compared to skin cues in the encoding of race.

## Data Availability Statement

The raw data supporting the conclusions of this article will be made available by the authors, without undue reservation.

## Ethics Statement

The studies involving human participants were reviewed and approved by the Macquarie University Human Research Ethics Committee (Code: 5201400561). The patients/participants provided their written informed consent to participate in this study.

## Author Contributions

KB conceived, designed, and coordinated the study, and helped draft the manuscript. DS participated in the design of the study, created the stimuli, collected the data, and helped draft the manuscript. OG aided in the design of the study, participated in data analysis, and drafted the manuscript. All authors gave final approval for publication.

## Conflict of Interest

The authors declare that the research was conducted in the absence of any commercial or financial relationships that could be construed as a potential conflict of interest.

## Footnotes

1 Students in the undergraduate participation pool were: 63% White European, 17% East/South East Asian, 7% South Asian, 7% Middle Eastern, 1% African, 3% mixed race (other groups < 1%). While the race of participants used in this study was not explicitly recorded, we have no reason to believe that their demographics differ substantially from those of the participant pool.
